# Freedom's values: The good and the right

**DOI:** 10.1111/theo.12431

**Published:** 2022-08-30

**Authors:** Pietro Intropi

**Affiliations:** ^1^ Trinity College Dublin Philosophy Department Dublin Ireland

**Keywords:** freedom, Hillel Steiner, moral status, non‐specific value, trust, value theory

## Abstract

How is freedom valuable? And how should we go about defining freedom? In this essay, I discuss a distinction between two general ways of valuing freedom: one appeals to the good (e.g., to freedom's contribution to well‐being); the other appeals to how persons have reason to treat one another in virtue of their status as purposive beings (to the right). The analysis of these two values has many relevant implications and it is preliminary to a better understanding of the relationships between freedom and justice. First, it contributes to shed light on the relationship between trust and the value of freedom, and on two attitudes towards freedom – promoting and respecting freedom. Second, it disambiguates between two versions of the claim that freedom has non‐specific/content‐independent value: one appeals to the good, the other to the right. And third, I show that certain implications concerning the definition of freedom follow from assuming an account of the value of freedom that exclusively appeals to the right, illustrating how the value of freedom can shape what freedom *is*.

## 1 TWO VALUES OF FREEDOM

How is freedom valuable? And how should we go about defining freedom? These are vexed questions in political philosophy, and it may be natural to think that one should first exactly define what freedom is and then discuss why freedom is valuable. In my view, it would be misleading to proceed this way. In Section 5, I show that certain implications concerning the definition of freedom follow from adopting a certain account of the *value* of freedom. That is, I argue for a value‐first approach to the conceptual analysis of freedom, according to which disagreements concerning how freedom (and unfreedom) should be defined are best understood in light of considerations concerning the value of freedom. Hence, questions concerning the value of freedom should be asked first: why is freedom valuable? What justifies attributing value to freedom? Typically, these questions are addressed within the framework of a discussion concerning whether freedom is (intrinsically) valuable as an end in itself or (extrinsically) valuable in virtue of contributing to other valuable goods (e.g., autonomy, human progress, well‐being) (e.g., Carter, 1999: Chap. 2; Kramer, 2017: Chap. 5). Recent contributions to the freedom literature have pointed to an additional aspect of the value of freedom that deserves careful study and that will be the object of my analysis in the first part of the article (Sections 1, 3). In Sections 1, 3, I examine two distinct ways of valuing freedom: one appeals to *the good*, the other to *the right*.^1^

In value theory and normative ethics it is commonplace to distinguish between the good, which pertains to the positive evaluation of outcomes and states of affairs, and the right, which pertains to how people should treat one another, and therefore to the moral evaluation of actions (Rawls, 1971: sections 5–6; Zimmerman, 2015). Consider how a state of affairs in which people have freedom can be valuable in virtue of axiological considerations concerning the good. For instance, it can be *good for* persons to have freedom because having freedom benefits persons (Carter, 1999: 50–54; Kramer, 2003: 430–431; Raz, 1986; Sen, 1992). Freedom can be valuable as

*’a good‐making property of people's lives*.*’* We are interested in measuring the degrees to which individuals are free, I thought, *because freedom is (for whatever reason) a good thing, either for the individuals who possess it or for society as a whole*. (Carter, 2009: 167, emphases added)

Ian Carter's quotation exemplifies a “Good‐Based View” – a way of interpreting the value of freedom that appeals to the good. As I claim in Section 2, there are many Good‐Based Views, and distinguishing between different senses of “goodness” of people's freedom contributes to clarifying the role that appeals to freedom's value can play in normative arguments. A second way of valuing freedom – I call it “The Status View” – does not directly appeal to the good but to the right. As Ralf Bader notes, this way of valuing freedom appeals to deontic reasons (concerning how persons have reason to treat one another) that prescind from well‐being and that are status‐based; in particular, such reasons count in favour of refraining from relevantly interfering with people's freedom in light of persons' status as purposive agents, as beings capable of setting ends (Bader, 2018: section 4). A number of freedom theorists appeal to status‐based considerations (e.g., Lawless, 2018; Olsaretti, 2005: 107).^2^ By way of illustration, consider Kant's notion of freedom as “independence from being constrained by another's choice” (Kant, 1996 [1797]: 393). Arthur Ripstein notes that for Kant, (equal) freedom:

[…] *is not a matter of people having equal amounts of some benefit*, however it is to be measured, but of the respective independence of persons from each other. (Ripstein, 2009: 33, emphasis added)

If the value of freedom is derived from a deontic reason – for instance, if freedom as independence is valuable – such a value does not consist in the presence of a benefit (Bader, 2018: 159), that is, in something that is *good for* the individual in virtue of contributing to her well‐being.^3^ And it is possible to attribute value to freedom by directly appealing to the right – to what persons owe to one another, or what they have reason to do – rather than to the goodness of having freedom (Garnett, 2018: 551–552).^4^ I examine The Status View in Section 3. Here I should clarify that, although I use the term “the right” (in opposition to “the good”) to indicate the distinctively normative domain of how people have reason to treat one another, The Status View does not necessarily derive freedom's value from a full‐blown *right to freedom* (e.g., from a right to Kantian freedom as independence); indeed, one could have a weaker reason – *some* reason (or a pro tanto reason) – to refrain from interfering with someone else's freedom, without such a reason's having the strength of a duty (correlative to someone's right to freedom).^5^ Indeed, I shall offer an interpretation of The Status View that includes an appeal to pro tanto reasons (Sections 3, 4).

Distinguishing between two values of freedom has many relevant implications for contemporary freedom debates. For instance, it justifies holding two distinct attitudes towards freedom – respectively, promoting and respecting freedom (Bader, 2018: 160). And, as I argue in Section 4, it allows to distinguish between two versions of the claim that freedom has *non‐specific/content‐independent* value (Carter, 1999; Kramer, 2003, 2017)^6^: one appeals to the good, the other to the right. Then in Section 5, I show how the definition of freedom (as mere absence of human interference) endorsed by Hillel Steiner can be justified exclusively in light of considerations concerning the right (i.e., in light of The Status View). Section 6 concludes. Before proceeding, some preliminary clarifications are in order. First, I broadly understand freedom as an opportunity concept (Taylor, 1979): the possibility to act or become, rather than as acting in particular ways (e.g., autonomously) or according to principles. Second, unless otherwise specified, I use the general term “freedom” to refer both to a “specific freedom” (to single opportunities to do certain actions *X* or *Y*) as well as to a degree of freedom (to freedom sets taken as a whole) (Carter, 1999). Third, some of the most debated questions in the freedom literature concern which types of obstacles are sufficient for describing someone as *unfree* (to do a specific action *X*). One aspect of such questions that I do not address in the paper is whether it is plausible to consider only obstacles that render *physically impossible* the doing of *X* as sources of unfreedom (Steiner, 1994: Chap. 2), or also obstacles that render the doing of *X* more difficult, costly, or less eligible (but not physically impossible) (Miller, 1983): I shall assume that at least the former type of obstacles is unfreedom‐generating, and leaving it open whether also obstacles of the latter type can be considered so.

## 2 GOOD‐BASED VIEWS

I wish to start with offering an analysis of different senses of “goodness” attributable to people's freedom. When theorists appeal to the goodness of having freedom, they often have in mind that freedom benefits persons, that it contributes to making people's lives go well (Carter, 2009: 167; Raz, 1986). As I clarify below, this entails attributing personal value to freedom, and I call this way of interpreting the value of freedom “The Personal Good View”.^7^ However, it should be noted that there are many interpretations of The Personal Good View (many Personal Good Views), and that there are also Good‐Based Views that do not attribute personal value to freedom. This clarifies a number of ways in which the claim that it is good to have freedom can figure as premise in arguments that prescribe to promote freedom or to distribute it according to certain principles.

### 2.1 Personal good views

Having freedom can be *good for* persons, it can make a positive contribution to individual well‐being and its components (e.g., autonomy). Interpreting the value of freedom this way means attributing *personal value* to freedom: it means asserting that people pro tanto benefit from freedom and that they have “an interest in freedom […]” (Carter, 1999: 33) insofar as freedom contributes to making their lives go well.^8^ I call this way of interpreting the value of freedom The Personal Good View. There are at least two dimensions alongside which we can distinguish between different versions of The Personal Good View. The first one concerns whether the (personal) value of freedom is *desire‐dependent*. On the desire‐dependent account, what makes it good for someone to have freedom is that the individual desires freedom. On such an account, a desire for freedom is a necessary condition for freedom's being good for someone. In contrast, on the desire‐independent account, having freedom is good for someone even if the individual does not desire freedom. This distinction can be illustrated with the following example: even if a monk is perfectly content with an ascetic life of meditation in a cell that cannot be opened from the inside (i.e., even if the monk has no desire for freedom), on the desire‐independent account the monk would benefit if someone opened the door of the cell, increasing the opportunities available to him. On such an account, there is a sense in which the monk's life would go better, even if the monk does not desire freedom, for example, having freedom can be said to make a positive contribution to the achievement of other valuable goods (e.g., autonomy, well‐being: Raz, 1986; see also section 4) that in turn make up a list of objectively valuable goods that have personal value.^9^

The second dimension concerns “who” can be said to benefit from freedom. Perhaps, the most intuitive way of interpreting The Personal Good View captures the idea that, if an individual *A* has freedom, the person who benefits from freedom is *A herself* (rather than someone else). But this is not the only way of spelling out The Personal Good View: on an alternative account, someone's freedom is valuable because it is *good for others* that the individual is free. Take *Prisoner*:

*Prisoner*: After detaining you in a prison cell for a year, I set you free. I do not wish to benefit *you* – in fact, I would want to keep you in prison for another year. The reason why I liberate you is because I wish to benefit my friend, who strongly desires that you are free.

Since it is good for you to have more freedom, by liberating you I (pro tanto) benefit you, but I also benefit my friend, fulfilling his desire to set you free, and it is good *for him* that you are free. So, your freedom has personal value also for my friend. Appeals to this type of value are not uncommon in the literature, and indeed they can figure in a consequentialist reasoning that implies to maximise people's freedom. For instance, consider Friedrich Hayek's views on freedom and progress. Hayek argues that maximising people's freedom is instrumentally valuable as a means to advancing human progress, which in turn can be considered valuable in personal terms (i.e., in virtue of benefiting persons); indeed, having greater opportunities (for cooperation) allows people to tread new paths and to experiment novel solutions to societal problems, and this in turn increases the likelihood of bringing about more innovation and progress (Carter, 1999: 46). Now, when it comes to explaining the benefits that freedom yields, Hayek claims that:

The benefits I derive from freedom are thus largely the result of the uses of freedom by others, and mostly of those uses of freedom I could never avail myself of. It is therefore not necessarily freedom that I can exercise myself that is most important for me. (Hayek, 1976 [1960]: 32)

So, for Hayek, it is good for *A* that others have (maximal) freedom, and indeed *A* mostly benefits from other people's freedom. In other words, on this interpretation of the relationships between freedom and progress, the fact that other people have freedom has personal value for *A*.^10^ I now want to show that there are also ways of understanding freedom's goodness that do not appeal to the benefits that people can derive from freedom (i.e., to the personal value of freedom). This will introduce the idea of impersonal value that plays a role also in the analysis of The Status View in the following sections.

### 2.2 Impersonal goodness

The value of freedom can be *detached* from the value that freedom has *for persons*; that is, freedom can have impersonal value. Freedom has impersonal value iff its value is not wholly reducible to well‐being. And it is impersonally good that persons have freedom iff freedom “contributes to the goodness of outcomes beyond the extent to which is good *for* people” (Temkin, 2003: 778). So, there are Good‐Based Views that interpret the value of freedom in impersonal terms.^11^ For instance, consider again the relationship between freedom and progress broached in the discussion of Hayek's views in the previous paragraphs. Although progress can be considered as having personal value, it can also have *impersonal* value. For example, one can think that the mere fact that humanity has reached a certain level of civilization (e.g., of economic, technological development) is valuable abstracting from the balance of harms and benefits attributable to such an achievement. And, if one agrees with Hayek that promoting people's freedom is a factor that causally contributes to bringing about more progress, a state of affairs in which people have maximal freedom can be *impersonally* valuable in virtue of contributing to the achievement of an impersonally valuable end (progress). Note that the achievement of an impersonally valuable end may clash with what is good for persons (i.e., with their well‐being). To illustrate this, one could think of a society in which people devote most of their time to the task of achieving as much progress as possible: for example, by working every day very long hours in order to make technological developments or scientific discoveries that contribute to progress. And that this in turn inevitably involves well‐being sacrifices (e.g., reducing the time for leisure or family life). Of course, a theory of justice that recognises the pull of achieving an impersonally valuable end should also have rules for balancing impersonal reasons with sacrifices in well‐being, unless (implausibly) the impersonally valuable end is deemed to be unconditionally valuable: that is, unless it is valuable no matter what are the consequences for persons (on unconditional value, see Carter, 1999: section 2.2).

## 3 THE STATUS VIEW

I now want to dig deeper into the analysis of The Status View. To recall, The Status View does not directly appeal to the goodness of having freedom but to deontic reasons concerning how persons have reason to treat one another in virtue of their status of agents: and such reasons prescribe to refrain from relevantly interfering with people's freedom (Section 1). In this Section, I offer an interpretation of The Status View that will emerge through a discussion of Matthew Kramer's understanding of deontic reasons to refrain from interfering with people's freedom in *Liberalism with Excellence* (Kramer, 2017). Kramer discusses the value of freedom in chapter 5, and one line of argument appeals to The Status View. In the course of introducing the distinction between the value/disvalue of doing *X* and the value of being free to *X*, Kramer notes that, even though *doing* something harmful for oneself (e.g., practicing self‐disembowelment) is obviously disvaluable, having the freedom to do so “is far from valueless” (Kramer, 2017: 200). For instance,

one's retention of the freedom to disembowel oneself is important because it is typically a hallmark of respect for one's deliberative maturity. In other words, the preservation of that freedom for any adult of sound mind bespeaks the fact that such a person is generally credited with sufficiently good judgment to reach appropriate decisions concerning self‐disembowelment as an option. To remove that option in order to prevent her from availing herself of it would be to evince a lack of trust in her inclination to arrive at suitable decisions on the matter. (Kramer 2017: 201; see also Kramer, 2003: 241).

Kramer's argument introduces considerations concerning (1) the value of freedom and (2) the disvalue of being deprived of the freedom to engage in self‐harm (the disvalue of unfreedom). As far as (1) is concerned, Kramer claims that having the freedom to engage in self‐harm “is important” – it has value – and that its preservation expresses recognition for people's “deliberative maturity” (which Kramer defines as a kind of “respect”). And that (2) removing such a freedom in order to protect people from engaging in self‐harm “would be to evince a lack of trust” in people's ability to decide for themselves how to act. The deontological nature of Kramer's argument is evident (see e.g., Kramer, 2017: 251–254) and I interpret Kramer's claims and others analogous statements in the book (e.g., Kramer, 2017: 211–212; 217) as appealing to The Status View: as pointing to an interpretation of the value of freedom that derives freedom's value from deontic reasons and status‐based considerations.

### 3.1 Clarifications about Kramer's endorsement of The Status View

I now want to examine the role of trust in The Status View. To that end, I should introduce some clarifications. First, although Kramer does not commit himself to a specific notion of trust, the relevant account of trust is within the broad family of trust‐as‐reliance views: in particular, *B* trusts *A* when *B* refrains from interfering with *A*'s freedom on the supposition that *A* will exercise her deliberative skills in a sensible manner with respect to which course of action is best for *A* (i.e., when *B* relies on *A*'s judgement with respect to which options *A* should choose).^12^ Second, note that, if *A* can be trusted to exercise his or her capacity for judgement with respect to any option (including also the option of whether to harm oneself), this gives reason to believe that freedom has what Kramer calls *content‐independent* value: that is, to claim that there is value in *any* freedom (including the freedom to harm oneself), irrespective of the content of the freedom in question, independently of whether it is the freedom to *X* or *Y* (Kramer, 2017: 200–207). Freedom's content‐independent value is an important aspect of freedom's value, and I'll get back to the implications of this point in Section 4.

Third, as evidenced by Kramer's quotation, The Status View introduces an interpersonal – or *relational* – dimension to the value of freedom: on The Status View, the value of *A*'s freedom is explained in terms of *B*'s reason to refrain from interfering with *A*'s freedom in light of *A*'s status of agent. Symmetrically, also the disvalue of unfreedom is explained in relational terms: as the disvalue generated by failing to abide by the deontic reason that grounds the value of freedom in the first place. Fourth, Kramer introduces his analysis of the value of freedom in the context of a broader discussion of how the state should treat its citizens (Kramer, 2017: Chap. 5–6).^13^ In particular, according to Kramer, when the state removes a citizen's freedom to engage in self‐harmful activities (e.g., the freedom to take recreative drugs) the state “treats [an individual] as if she cannot be trusted to look after her own good” (Kramer, 2017: 212). And for this reason such freedom removals are always wrong (Kramer, 2017: 255–256). Fifth, an implication of Kramer's views is that a reason to refrain from depriving someone of a relevant freedom (to engage in self‐harm) subsists only on condition that the individual can be trusted to judge in a sensible manner which option is best for herself, and therefore only insofar as the individual possesses the relevant cognitive and volitional skills at a sufficient level (Kramer, 2017: 227; 233).^14^ Henceforth, I leave aside the discussion of cases in which this condition does not obtain.

### 3.2 Trust and The Status View

Kramer is right that if one is deprived of a certain freedom (the freedom to harm oneself) “*in order to prevent her from availing herself of it* would be to evince a lack of trust in her inclination to arrive at suitable decisions on the matter” (Kramer, 2017: 201, emphasis added), and that therefore lacking freedom in such cases is disvaluable. But, of course, a certain freedom could be removed without intending to protect someone from self‐harm. Consider the following example:

*Quicksand*: Mildred is an artist, and she encloses a quicksand area with electrifying wire exclusively because she wants to create an open‐air installation, rather than in order to deprive Noah of the opportunity to walk into the quicksand.

Some preliminary clarifications are in order. Note that *Quicksand* – like the case of self‐disembowelment mentioned above – concerns the removal of an opportunity to commit suicide; that is, I assume that Noah would terminate his life if he were to walk into the quicksand. And I take it that the constraint – the fact that the area is enclosed with electric fences – is of the type that can generate unfreedom because it would make it physically impossible for Noah to walk into the quicksand, for example, because touching the electric fence would kill, rather than merely deter, Noah (Section 1).^15^ I also assume that Noah has no claim right to walk into the quicksand (nor that it is impermissible for him to do so), and so that Mildred does not infringe upon Noah's rights by enclosing the area. Now, is there any disvalue in the fact that Noah is deprived of the opportunity to commit suicide (by walking into the quicksand), *on deontic grounds*? If we assume an account of The Status View that is tied exclusively to trust considerations, such an account cannot recognise that there is some freedom‐related disvalue in Noah's predicament since Mildred is not motivated by lack of trust in Noah's deliberative capacities: she encloses the area exclusively for artistic purposes rather than in order to protect Noah. However, I think that it is possible to offer an interpretation of The Status View that recognises that there is some freedom‐related disvalue in Noah's predicament.^16^ In what follows, I develop such an interpretation, drawing on Carter's and Kramer's reflections on freedom and agency.

### 3.3 Agency and The Status View

I now want to recast Carter's and Kramer's analysis of the relationships between freedom and agency in deontic terms in order to explain how there can be freedom‐related disvalue in Noah's predicament according to The Status View. One preliminary clarification is in order. Since The Status View grounds the value of freedom (and the disvalue of unfreedom) on a reason to refrain from interfering with someone else's freedom, we are looking for such a reason. The *strength* of the reason in question does not need to be that of an *all things considered* or *decisive* reason: it can be a contributory or *pro tanto* reason, where I define a pro tanto reason as a reason that has some weight and that counts in favour of doing or omitting to *X* (of refraining from interfering with Noah's freedom) without necessarily determining an overall judgement over whether Mildred should do/omit to *X* (since such a judgement is determined by the right balance of all reasons that apply to the case).^17^ And therefore, if there is some reason for Mildred to refrain from interfering with Noah's freedom – and such a reason can be explained in status‐based, deontic, and relational terms – it will be possible to ground The Status View on such a reason.

Consider how Carter (1999: 42–43; 58–59) and Kramer (2017: 204–205) interpret the relationships between freedom and agency, discussing an influential article by Thomas Hurka (Hurka, 1987). The views of these theorists can be reconstructed as follows: when someone is deprived by someone else of the freedom to *X* (e.g., when Mildred removes Noah's freedom to walk into the quicksand), someone is deprived of an opportunity for agency, not just because one is deprived of the opportunity to *do X* 18 but also because one is deprived of the opportunity to *refuse* to *X* in a context in which it would be possible to *X* (i.e., to do otherwise). This is in turn disvaluable because it subtracts to “the declinatory force of a person's choices” (Kramer, 2017: 204).^19^ Drawing from Hurka (1987: 362–63; 366–371), Carter explains this point by referring to the value of agency, to the value of being able “to make an impact on the world, by intervening in the causal chains which bring about events and states of affairs” (Carter, 1999: 42). And one's potential for agency is diminished when freedom is diminished: when one lacks the freedom to *X*, one's refusal to *X* cannot make any difference on how the events would unfold anyway (since one would in any case be unfree to *X*). Hence, one reason why depriving one of certain freedoms is disvaluable is because it diminishes the potential of an individual's *refusals* (to act in certain ways) to make an impact on the course of the events (Carter, 1999: 42–43).^20^

Given the taxonomy of different kinds of values presented in this and the previous sections (Sections 1, 3), it is possible to interpret the value of freedom described above in many ways.^21^ One is the personal good interpretation (Section 2): on this understanding, it is *good for* an individual to not being interfered with since this increases the individual's potential to make an impact on the world, and insofar as personal value is attributed to individual agency. That is, on this view being able to make an impact on the world can be said to positively contribute to individual well‐being: it is something that contributes to make people's lives going well. Second, it can be *impersonally* good *that* people have more opportunities (rather than *good for* persons) (Section 2). This understanding is premised on the idea that impersonal value is attributable to the existence of beings endowed with agential capacities. And, I take it, if impersonal value is attached to the existence of such beings in virtue of their agential capacities (i.e., in virtue of their status as agential beings), there is also impersonal value in the fact that such agential beings have opportunities to exercise their agency. And third, the value of Noah's freedom can be understood in *relational* and *impersonal* terms, and this interpretation captures the value expressed by The Status View. If it is impersonally good that agential beings have opportunities, this grounds a reason for *others* to refrain from interfering with such opportunities. This is because such agential beings have (at least some) reason to refrain from compromising the value in question. And, given that such a reason is derived from considerations concerning people's status of agents, rather than from well‐being, it is impersonal (rather than personal). So, I interpret The Status View as expressing the impersonal value of a *relationship* in which *A* refrains from relevantly interfering with *B*'s opportunities. And the value expressed by The Status View is *relational* and *impersonal*.^22^ Furthermore, I have claimed that the reason that grounds The Status View can be a *pro tanto* reason: a reason that has some weight when deliberating about how to act, but that does not conclusively entail that one ought to refrain from interfering with someone else's freedom.^23^ There are a number of reasons why the analysis of The Status View offered in this section matters. In particular, it allows to distinguish between two ways of interpreting the idea that freedom has content‐independent (non‐specific) value (Section 4). And this in turn has implications for understanding the role of freedom in a theory of justice. In Section 5, I offer an interpretation of the role of freedom in Steiner's theory of justice: I shall argue that, although Steiner does not claim that freedom is *good for* persons (i.e., that freedom has personal value), his theory of justice can be interpreted as implicitly assuming an account of the (impersonal and relational) value of freedom according to The Status View. Hence, I take Steiner's theory to show the possibility of appealing to freedom in a theory of justice without appealing to freedom's *goodness for* persons (i.e., to people's interest in freedom).^24^

## 4 TWO ATTITUDES TOWARDS FREEDOM AND TWO TYPES OF NON‐SPECIFIC/CONTENT‐INDEPENDENT VALUE

In this section, I want to illustrate two relevant implications that distinguishing between different ways of valuing freedom (Sections 1, 3) has for contemporary freedom debates. First, I discuss two attitudes towards freedom: promoting and respecting freedom (Bader, 2018: section 4). And, second, I argue that there are two general ways of interpreting the claim that freedom has non‐specific (Carter, 1999)/content‐independent (Kramer, 2003, 2017) value.^25^

### 4.1 Promoting versus respecting freedom

According to Thomas Scanlon “[u]nderstanding the value of something is not just a matter of knowing *how valuable* it is, but rather a matter of knowing how to value it – knowing what kind of actions and attitudes are called for” (Scanlon, 1998: 99). So, what attitude(s) should we have towards *freedom*? What is the appropriate way of responding to the value of freedom? This question bridges the evaluative dimension of attributing value to freedom with the normative dimension of establishing what should be done with people's freedom. Therefore, addressing it contributes to clarifying the role that the value of freedom can play in normative arguments, especially in a theory of justice. As Bader has pointed out, (1) attributing value to freedom in virtue of axiological considerations (i.e., in virtue of considerations concerning the good) entails a reason to *promote* freedom (e.g., to increase it [up to a satiating point] or to maximise it), and (2) attributing value to freedom in virtue of deontic reasons (The Status View) entails a reason to *respect* freedom – that is, to refrain from compromising such a value by relevantly interfering with people's freedom (Bader, 2018: 159–160). I want to clarify this further by elaborating on Philip Pettit's remarks on Stanley Benn's distinction between “value‐centred” versus “person‐centred” reasons (Benn, 1988), which I take it to mirror the distinction between good‐based and right‐based considerations:

The value‐centred reason directs my attention to a property, usually a universal property, which is hailed as desirable and worth promoting […]. The person‐centred reason, however, […] does not direct me in the first place to a property I should promote but rather *to an action I should perform*: I should not interfere with this person, I should give him equal consideration, or whatever. The value‐centred reason directs me also of course to an action I should perform but it does so via directing me to a feature I should promote. The person‐centred reason directs me to an action I should perform without specifying such a property to be promoted. (Pettit, 1989: 123, emphases added)

Applying Pettit's considerations to the value of freedom clarifies that *Good‐Based Views* (value‐centred reasons) interpret the value of freedom directly in terms of the value that freedom has as a (spatio‐temporal) *physical property*: as opportunity to use physical objects within determined portions of space and time (Carter, 1999; Kramer, 2003). Having such a property is valuable insofar as it contributes to outcomes deemed to be good, for instance, insofar as it contributes to the attainment of well‐being or societal progress (Section 2). And so, to the extent that the (personal or impersonal) goodness of the outcomes gives us reason to promote them, we also have reason to promote freedom. By contrast, The Status View attributes value to freedom *directly* in virtue of an *omission to act* – in virtue of a deontic reason to refrain from relevantly interfering with people's freedom – and indirectly as a physical property.^26^ In other words, on The Status View there is impersonal value in the physical property “freedom” (as spatio‐temporally defined opportunity to use certain objects) in virtue its *extrinsic relational* properties, that is, because having freedom denotes in spatio‐temporal terms that a valuable relationship subsists between two persons who refrain from interfering with one another's freedom. So, on The Status View the appropriate attitude towards freedom is to *respect* it.^27^

### 4.2 Two types of non‐specific/content‐independent value

Distinguishing between two general values of freedom allows to distinguish between two ways of interpreting the claim that freedom has *non‐specific/content‐independent* value: one appeals to the good, the other to the right. In my view, this point has not received enough attention in the literature, and I want to bring it out more fully in this context. “Non‐specific” (Carter, 1999) and “content‐independent” (Kramer, 2003, 2017) capture the same aspect of the value of freedom: the idea that having freedom has (some) value independently of its content, irrespectively of what freedom allows one to do (Carter, 1999; Kramer, 2003: 240–245).^28^ Carter defines non‐specific value as follows:

If freedom is ‘non‐specifically valuable’, then we attach value to our freedom not only because of the specific things it allows us to do, but also because of the mere fact of our having freedom. To say that freedom is non‐specifically valuable is to say that it is valuable ‘as such’. (Carter, 1999: 34)

For Carter what is non‐specifically valuable is (having) *a certain degree* or *a measure of freedom*; that is, Carter's notion of non‐specific value applies to what Carter calls “overall freedom” (the total amount of the good “freedom” that an individual possesses) (Carter, 1999: Chap. 1). Analogously, one can also say that the value identified by Carter in the quotation above can also be applied to *specific* freedoms (the freedom to do specific things *X* or *Y*). One can say that having a specific freedom to do something *X* is valuable irrespectively of its content – irrespectively of being the freedom *to X*: that is, that it is valuable qua instance of the good “freedom” (qua freedom). This can be captured by Kramer's notion of content‐independent value (Kramer, 2017: Chap. 5). What matters is that, if freedom (both single specific freedoms and a measure of freedom) has that type of value, then freedom is also valuable *qua freedom*: it is valuable not just in virtue of its specific content but also qua freedom. And claiming that freedom has non‐specific/content‐independent value implies asserting that *any* specific freedom (any freedom to *X* or *Y*) and freedom set has (some) value in virtue of partaking to the good “freedom.”

With these clarifications at hand, I now want to show that there can be two ways of interpreting the claim that freedom has non‐specific/content‐independent value. I illustrate this point by, first, considering ways of understanding non‐specific/content‐independent value that appeal to the good, moving then to the analysis of an account of non‐specific/content‐independent value that appeals to the right (to deontic reasons). Let's start with good‐based versions of non‐specific/content‐independent value: especially, consider the interpretation that appeals to personal value. For example, Carter argues that people not only have an interest in certain *specific* (valuable) freedoms (e.g., the Rawlsian “basic liberties” or a range of capabilities to function well [Rawls, 2005; Sen, 1992]), they also have “an interest in freedom *as such*” (Carter, 1999: 33). Indeed, having a degree of freedom as such can be said to contribute to well‐being (subjectively interpreted as desires satisfaction) and autonomy (as a component of well‐being [Raz, 1986]):

*Desires (Subjective Well‐being)*: Since there is an ineliminable degree of uncertainty about the nature of our future ends, having (more) freedom as such contributes instrumentally to the attainment of our desires. Indeed, not knowing with certainty which *specific* freedoms will better serve our future ends, “we can only be sure (or as sure as possible) of attaining the ends liberty serves by making sure that there is as much liberty as possible (at least *ceteris paribus*)” (Carter, 1999: 51–52; see also Kramer, 2017: 205–206).

*Autonomy*: Having freedom as such is a constitutive component of autonomy, understood as the ideal of shaping one's own life according to one's own evaluative judgements. Having (more) freedom as such – including the freedom to choose and act otherwise – allows one to exercise greater control over the direction one's own life should take, giving the opportunity to decide *whether* to act in a given way while at the same time having the opportunity to do otherwise (see Kramer, 2003: 431–432; 2017: 207; Carter, 1999: 59–60).

According to *Desires* and *Autonomy* having a degree of freedom as such – having freedom non‐specifically – *benefits* persons.^29^ Moreover, since appealing to the good entails a reason to promote freedom, attributing personal non‐specific/content‐independent value to freedom entails a reason to promote freedom *non‐specifically* – that is, to promote people's degree of freedom *as such* (at least up to a satiating point) rather than only certain specific freedoms. Then, consider the deontic interpretation of non‐specific/content‐independent value. Such a view does not directly appeal to the good but to the right:

*Non‐Specific/Content‐Independent Value and Deontic Reasons*: Freedom has non‐specific/content‐independent value because there is a reason (pro tanto or all things considered) to refrain from interfering with *any* freedom (with any specific freedom or with a degree of overall freedom), irrespective of the content of the freedom(s) in question.

On this version, attributing value to freedom non‐specifically/in a content‐independent way amounts to claiming that there is reason against interfering with *any* freedom (or freedom set), irrespective of content. This faces the challenge of explaining how there could be reason to refrain from interfering with people's freedom to perform morally impermissible actions (especially, severely wrong actions). For instance, as Bader claims:

As soon as the freedom to torture, to rape, and to murder classify as genuine freedoms […], it is evident that considerations of freedom do not as such constitute pro tanto reasons. (Bader, 2018: 157)^30^

In my view, Bader overlooks an important aspect of how the value of having opportunities can be derived from deontic reasons. Of course, the fact that someone is disposed to perform a morally impermissible action can be sufficient for justifying preventing the individual in question from acting wrongly, thereby also depriving the individual of the opportunity to do so. Especially, when we consider the actions mentioned by Bader (torturing, killing, raping), it is morally obligatory for the state to use force in order to prevent someone from actualising her intentions to kill, torture, and rape.^31^ However, when one has *no intention* of acting wrongly, I contend that there is *some* reason to refrain from depriving one of the opportunity to act wrongly.

In ideal theory we take people's dispositions to act according to the requirements of morality and justice as parametric – that is, we assume “strict compliance” (Rawls, 2001: 13) with moral principles.^32^ And, applying the arguments developed in Section 3 to the discussion of opportunities to perform morally impermissible actions, there are at least two ways of justifying why under such conditions there is some reason (a pro tanto reason) to refrain from relevantly interfering with people's opportunity to perform morally impermissible actions: First, removing people's opportunities to act immorally has (impersonal) disvalue in virtue of diminishing the potential of people's choices (to act morally) to have an impact on the world; indeed, when one is deprived of the opportunity to act immorally, the choice of refusing to act immorally does not make a relevant causal contribution to the course of the events (since in any case one would have been unable to do otherwise, to act immorally). And, as we have seen in Section 3, this grounds a reason to refrain from interfering with people's opportunities. Second, to the extent that refraining from interfering with people's opportunities to act immorally can be understood as a behavioural component of expressing recognition of others as *moral* agents – as beings who recognise the binding force of moral principles – refraining from interfering in turn contributes to establish a relationship of trust between agents who are committed to act morally. Hence, I have shown that it is plausible to interpret The Status View *non‐specifically/in a content‐independent way*, which implies asserting the existence of a (pro tanto) deontic reason to refrain from relevantly interfering with any opportunity (including the opportunity to perform morally impermissible actions), irrespective of content. And, as we have seen, my argument gestures towards a possible role that this way of interpreting The Status View can play in normative arguments. In particular, if we assume a perspective in which people are motivated to act according to moral principles (ideal theory), people have reason to refrain from interfering with one another's freedom (or opportunities) *as such*, and such a reason should be considered part of the correct moral theory or theory of justice.

## 5 THE DEFINITION OF FREEDOM AND THE STATUS VIEW

I now want to bring the discussion of two ways of valuing freedom to bear on questions concerning the definition of freedom: that is, concerning the conditions under which someone can be described as free (or as lacking the freedom) to *X*. So far, I have assumed that freedom should be understood as an opportunity concept (Section 1), but of course there are many *conceptions* of freedom as opportunity. So, how should we go about defining freedom? In particular, should the value of freedom play any role in the definition of freedom? In what follows I endorse Dworkin's view that ethical‐political concepts (among which, freedom) are interpretive (Dworkin, 2011: Chap. 7–8 and 17), which means that disagreements about their *definition* are best understood in the light of considerations concerning their *value*.^33^ Hence, we cannot clearly define what freedom *is* without assessing why we value freedom. In particular, I see the interpreter's job as that of finding definitions that *fit* with a given understanding of the value of a concept, and also with interpretations of other relevant values (e.g., justice).^34^ Now, consider a possible objection to this interpretive method: since we may find that freedom is valuable for a number of different reasons – and in turn such reasons point to connections between freedom and a plurality of other values (e.g., agency, well‐being, democracy) – one may think that the interpretive approach could leave us with no clear indication of what is the best definition of freedom. Or, worse, that we may end up with an incoherent definition since a certain reason to value freedom may be conducive to define freedom in a certain way, while another reason may lead us to define freedom differently.^35^ Although I cannot fully address this general worry here, I wish to clearly demarcate the ambitions of the following arguments. First, I see it as a positive implication of the interpretive method that it prompts to seek coherence between the definition of freedom and reasons to value freedom. Second, I shall not take a stance on whether the definition of freedom should be interpreted in light of *every* reason to value freedom.^36^ In my view, to offer a coherent argument, it is enough that the interpreter shows that a certain definition can be derived from a selection of reasons to value freedom, in particular, from those reasons that connect freedom to other values that make up a coherent normative system, for example, a theory of justice. By doing so, the interpreter puts forward reasons to define freedom in a certain way by appealing to how the value of freedom is integrated in a wider network of values (e.g., in a theory of justice). More specifically, in this section I wish to show that certain aspects of the definition of freedom can be interpreted in light of an account of the value of freedom that exclusively appeals to deontic reasons (The Status View) in the context of a theory of justice that does not appeal to well‐being (i.e., to freedom's goodness *for persons*).^37^ To do this, I should introduce questions concerning the relationships between freedom and ability, which are widely discussed in the literature. In particular:

*Q1*: Should freedom be understood as mere absence of constraints attributable to other human beings, or as (also) requiring the ability to act?^38^

For instance, suppose that nobody would stop me from swimming across a lake, though “my lungs or my limbs would give in before reaching the other side” (Van Parijs, 1995: 23), am I really free to do it? Is the fact that nobody else relevantly interferes with my opportunity to perform a certain action sufficient for freedom, or does freedom require also the ability to act? Some theorists claim that to be free to *X* one needs to be able to *X* (in the absence of the relevant human interference); for these theorists, freedom is ability‐based (Kramer, 2003; Sen, 1992; Van Parijs, 1995). Other theorists endorse definitions of freedom according to which one can be free to *X* even without being able to *X* (Berlin, 2002; Miller, 1983; Steiner, 1994), as long as one is not relevantly interfered with *by others* (Berlin, 2002: 169; Miller, 1983: 70). Support to the latter view comes from endorsing what I call “Relational Freedom”:

*Relational Freedom*: An obstacle is unfreedom‐generating if its existence is in the relevant sense attributable to other human beings.^39^

Whether one should endorse Relational Freedom depends on how one should answer the following questions concerning the *origin* (human or natural) of the obstacles that can be considered as unfreedom‐generating (Carter, 1999: section 8.1):

*Q2*: Should only obstacles that are in the relevant sense attributable to other human beings be considered as unfreedom‐generating or also natural obstacles (e.g., physical disabilities, psychological incapacitations, natural disasters)?^40^

The aim of what follows is to show that, if one endorses The Status View (i.e., if one endorses the relational understanding of freedom's value introduced in Section 3), one has reason to endorse Relational Freedom – that is, to assume that only constraints in the relevant sense attributable to other human beings can be unfreedom‐generating – and to interpret freedom as mere absence of human interference.^41^

Consider the definition of unfreedom. To recall, on The Status View the value of freedom is *relational* (Section 3). And this commits one to the view that freedom's value is compromised when others relevantly interfere with *A*'s opportunities. Hence, this justifies also defining unfreedom relationally: it justifies assuming that a necessary condition for ascriptions of unfreedom is that a constraint is in the relevant sense attributable to other human beings. In other words, the endorsement of a certain account of the value of freedom (The Status View) has implications for how unfreedom should be defined: it justifies endorsing Relational Freedom. This point and the interplay between the definition of unfreedom and status‐based considerations is noticed by a number of authors.^42^ Now, as far as the definition of *freedom* is concerned, which conceptual implications can be derived from The Status View? I address this question with reference to Hillel Steiner's understanding of the relationship between freedom and justice.

According to Steiner, freedom is a *bivalent* concept: that is, for Steiner an individual *A* can be *either free or unfree* to *X and tertium on datur*. That is, if *A* is not free (to *X*), she is necessarily unfree (Steiner, 2001). Hence, on this account, freedom amounts just to *lack of unfreedom*. Second, Steiner also endorses Relational Freedom (Steiner, 1983: 74–75; 1994). Hence, since for Steiner freedom is bivalent and “a social relation, a relation between persons” (Steiner, 1983: 74), one should be considered free as long as there are no relevant obstacles imposed by other human beings. Therefore, Kramer claims that Steiner “classifies mere inabilities as freedoms” (Kramer, 2003: 361): that is, that Steiner classifies someone who is physically unable to *X* as *free* to *X* insofar as such an inability is not in the relevant sense attributable to human beings. Now, Steiner interprets the relationships between freedom and justice in deontic terms, as illustrated by the following quotation:

We unavoidably restrict one another's freedom. And justice is about how those restrictions ought to be arranged. What it's *not* about are the ends which might be achieved by that arrangement. Questions of justice arise precisely where the moral permissibility of one person's restricting another's freedom is not determined by the comparative merits of the ends to which they are respectively committed. (Steiner, 1994: 1‐2)

A general remark about Steiner's methodology is necessary: according to Steiner, a theory of justice can be established simply by means of conceptual analysis and “by examining the formal or characteristic features of rights” (Steiner, 1994: 2).^43^ Hence, as Carter observes, Steiner does not need to claim that freedom is *valuable* “in order to say what justice is” (Carter, 2009: 172). Certainly, Steiner prescinds from assuming that freedom has personal value. Steiner's main substantive conclusion in *An Essay on Rights* – that justice assigns to each individual equal freedom (Steiner, 1994: 208–230) – does not appeal to the personal value of freedom: the right to equal freedom (Steiner, 1974) – and, hence the original rights through which equal freedom is instantiated (Steiner, 1994: Chap. 7–8) – does not entitle to a *benefit* that makes people's lives going well, it entitles to an equal degree of *unpreventedness* (lack of restrictions on one another's freedom) requiring that one is “no *more* prevented than others” (Steiner, 1994: 226). Now, even granting that Steiner can do away with any claim about freedom's value, consider how his definition of freedom fits with interpreting justice purely in deontic terms: since justice is about arranging mutual restrictions on people's freedom, the definition of freedom that serves the purpose of this understanding of justice is one that interprets freedom in terms of lack of human interference (Relational Freedom). And such a definition can be *interpreted* in light of the “intrinsic and non‐derivative value of justice” (Steiner, 1994: 1). Second, it is actually debatable whether Steiner can do away with any appeals to freedom's value: in my view, Steiner implicitly assumes The Status View when he appeals to the Kantian prescription to treat others never merely as means as justification of the equal freedom rule. As Steiner claims, such a prescription:

is generally interpreted as requiring one to *respect the agency of others* by performing no action that *subordinates their sets of purposes* to one's own […]. Since one's freedom is a necessary condition of one's pursuing any purposes at all, actions that diminish one's freedom to less than that enjoyed by their perpetrators are pretty strong candidates for being described as actions subordinating one's purposes to theirs. (Steiner, 1994: 221 emphasis added)

The edifice of Steinerian justice rests on a freedom‐based interpretation of appropriately responding to persons' (equal) moral status as agential beings. And this implies assuming that freedom has value, according to The Status View. First, since for Steiner justice requires to refrain from compromising the (original) equal distribution of freedom, such a distribution of freedom has relational value: it establishes a relationship that is valuable in virtue of a (deontic) requirement of justice. Hence, also the specific freedoms assigned by such a distribution have relational value, in virtue of an all things considered reason to refrain from upsetting the (original) egalitarian distribution of freedom. So, Steiner implicitly appeals to an interpretation of The Status View grounded on (all things considered) reasons to refrain from compromising people's equal freedom. Furthermore, Steiner can be interpreted as also appealing to *pro tanto* (deontic) reasons. Indeed, if, following Steiner, one considers the fact that “we unavoidably restrict one another's freedom” (Steiner, 1994: 1) as the fundamental problem of justice – thereby interpreting justice as being about how (restrictions on people's) freedom should be fairly distributed – this must be because it is implicitly assumed that in the original situation in which no distributive rule is in place there is both some reason (a pro tanto reason) to refrain from interfering with one another's opportunities as well as some reason to *exercise* one's own agency (thereby limiting the freedom of others). Hence, Steiner can also be interpreted as appealing to pro tanto deontic reasons.

Thus, consider how Steiner's bivalent definition of freedom as mere absence of human interference can be interpreted in the light of Steiner's implicit assumption of The Status View. If freedom's value is understood exclusively in terms of absence of human interference, nothing compromises it once interferences are lifted, and this justifies describing *A* as *free* to *X*, even if (physically) unable to *X*, as long as others do not relevantly interfere with *A*'s opportunity to *X*: it justifies bivalence. Consider a possible objection to this interpretive reasoning: one can maintain that it is possible to believe (1) that freedom is just the absence of human interference (Steiner's bivalence) while also claiming (2) that there is *no value* in having certain freedoms (e.g., in the freedoms to perform the actions that one is in any case unable to do, absent human interference). Though there is no contradiction in holding (1) and (2), in my view this objection misfires: indeed, if freedom's value is understood in terms of absence of human interference (The Status View), one is *drawn* to interpreting freedom as *non‐specifically/content‐independently valuable* (on deontic grounds) (Section 4): that is, one is drawn to conclude that every opportunity has some value *qua freedom* – including the opportunities to perform actions that one would not be able to do – insofar as such opportunities are not relevantly obstructed by others.

## 6 CONCLUSION

In this paper I have shown that distinguishing between two general ways of valuing freedom (one appeals to the good, the other to the right) has many implications for contemporary freedom debates. For example, there are two ways of interpreting freedom's non‐specific/content‐independent value: while freedom theorists often appeal to the claim that it is non‐specifically *good* (for persons) to have freedom, it is also plausible to believe that freedom has non‐specific/content‐independent value on deontic grounds, especially if in ideal theory we assume that people are fully motivated to comply with moral principles. Moreover, the analysis of two attitudes towards freedom (promoting and respecting freedom) can be seen as bridging the evaluative with the normative, and as leading to questions concerning the role of freedom in a theory of justice. Indeed, the theme of the relations between freedom and justice underlies also the discussion of which definition of freedom can be interpreted in light of The Status View. And I have argued that the definition of freedom that fits with Steiner's deontological approach to justice is one that interprets freedom as mere absence of human interference.

## FUNDING INFORMATION

This project has received funding from the European Research Council (ERC) under the European Union's Horizon 2020 research and innovation programme (grant agreement No 819043). Some of the work incorporated in this paper draws from the research of my doctorate, which was funded by The Clarendon Fund (Clarendon Scholarship), by Nuffield College, Oxford (Nuffield College Graduate Scholarship), and by The Institute for Humane Studies (Summer Research Fellowship and PhD Scholarship).

## Footnotes

^1^
The distinction between two values of freedom is highlighted by Ralf Bader in §4 of Bader (2018): for instance, Bader claims that on his moralised understanding of freedom “the significance of freedom is understood not axiologically but deontically” (Bader, 2018: 160). See also Garnett (2018: 551–552); Olsaretti (2005: 107).

^2^
See also Pettit's analysis of the relationships between (robust) non‐interference and respect for persons (2015: chap. 3). On Pettit's account, status‐based considerations appeal also to the good (Pettit, 2015: chap. 7). In this context, I leave the discussion of Pettit's complex views aside. See also Axel Honneth's recognition‐based understanding of freedom (Honneth, 2014), where different freedom domains – negative freedom as non‐interference, “reflexive freedom” as autonomy, and the “social freedom” that people should have as members of a society (e.g., qua consumers, workers, or democratic citizens) – are realised through or constituted by intersubjective practices of recognition (e.g., in the labour market social freedom is obtained when, inter alia, society adequately recognises the dignity of workers (Honneth, 2014: 223–253)).

^3^
Note that Ripstein's quotation can also be taken to imply scepticism about the possibility or relevance of measuring freedom. I leave the discussion of freedom's measurement aside in this context.

^4^
It is also possible that the value of freedom is composite and draws on both axiological (good‐based) and deontic (right‐based) considerations. In this context, I leave this point aside.

^5^
An anonymous reviewer asks me to clarify whether in the case in which The Status View derives freedom's value from a full‐blown right to freedom the value of freedom resides (1) in the good of having the right itself or (2) in the fact that the right is not infringed upon. An implication of asserting (1) would be that there is value in the formal recognition of the right (e.g., when it is formally recognised in the law) even if in fact the right is systematically violated. For example, a racial minority could have certain rights formally recognised by the law, whilst also having such rights de facto infringed upon by members of the majority who act with the complacency of the public authorities (I owe this example to the reviewer). In contrast, one may worry that asserting (2) (i.e., claiming that the freedom's value resides in the fact that the freedoms of the right‐bearers are not interfered with) implies reducing freedom's value to the good (of non‐interference), and hence ultimately reduces The Status View to a kind of Good‐Based Views. I think we should endorse (2), and that this does not imply a reduction of The Status View to a Good‐Based View: what is claimed by The Status View is simply that there is value in freedom (e.g., as non‐interference) *in virtue of* a deontic reason (i.e., that what grounds the goodness of freedom is such a reason), and that not interfering with someone else's freedom is the behavioural aspect of fulfilling the status‐based reason. So, insofar as it is possible to justify the value of freedom by appealing directly to such a reason (rather than to the goodness of having freedom), The Status View is not reduced to a Good‐Based View. I thank the reviewer for asking to clarify this point, and for illustrating the implications of possible ways of addressing this problem.

^6^
If freedom has non‐specific/content‐independent value, it is valuable independently of what it allows to do (§4).

^7^
I owe this formulation to an anonymous reviewer.

^8^
More precisely, Carter's claim is that people have an interest in a measure of freedom *as such*, not just certain in certain specific freedoms. If people *pro tanto* benefit from freedom, having (more) freedom is *to that extent* good for persons, though overall one may be better off by having *less* freedom (see Carter, 1999: 38–40).

^9^
On the distinction between desire‐fulfilment and objective list theories of personal value, see Fletcher, 2016: chap. 2–3. Obviously, it can be perfectly reasonable for the monk to sacrifice freedom for other objectively valuable goods (e.g., a life of contemplation) since on a pluralist view freedom is only one of many objectively valuable goods.

^10^
A reviewer notes that Hayek should be interpreted as claiming that what has instrumental value for the achievement of progress is that people's degrees of overall freedom are expanded (where “overall freedom” is the total amount of freedom possessed by each individual), which does not commit Hayek to the view that such a value is attributable to any specific freedom (to any freedom to perform specific actions *X*, *Y*, …), for example, to the freedom to set off a bomb that would destroy humanity. On the distinction between specific and overall freedom, see (Carter, 1999: chap. 1). Note, moreover, that there are also aggregative interpretations of The Personal Good View; for instance, if progress is understood as a “a social goal” (Carter, 1999: 46) – something for collectives to aim at – the beneficiaries can be said to be individuals as members of a given society, and it is good for them *in the aggregate* to have maximal freedom.

^11^
Note that also on The Status View the value of freedom is impersonal; but on such a view, the impersonal goodness attributed to freedom is derived from a deontic reason (see §§3–4).

^12^
Kramer's adoption of this account of trust is evident, for example, in the discussion of the value of being free to consume hallucinogens (Kramer, 2017: 211–212). On the relationship between trust and reliance, see Goldberg, 2020. Baier (1986) is the locus classicus for accounts of trust that appeal to reliance.

^13^
Especially in order to rebut versions of perfectionism (edificatory perfectionism) that justify policies that deprive people of the freedom to engage in self‐harmful activities (e.g., taking recreative drugs) for the sake of bettering people's lives.

^14^
The acknowledgement of this point is not problematic for Kramer: refraining from relevantly interfering when people can be trusted to judge by themselves what is good for them “is obviously consistent with one's recognizing that some human adults on some occasions are decidedly not of sound mind” (Kramer, 2017: 227), and that therefore “in extreme cases” (e.g., “where people are acting or seeking to act self‐destructively while in the throes of mental illness or inebriation or hallucinations or drug addiction, or where people are imperilling themselves because they harbour seriously mistaken beliefs about the specificities of the situations that confront them […]” (Kramer, 2017: 227)) it is legitimate for the state to intervene. The exact range of such cases is debatable and this point is not addressed in Kramer's discussion.

^15^
Note that, on Kramer's account, the fact that Mildred does not deliberately *intend* to deprive Noah of the relevant opportunity is irrelevant to judge whether Noah can be said to suffer an unfreedom since for Kramer the intentions of the interfering agent do not affect unfreedom ascriptions: what matters is exclusively that the interfering agent's actions impact on someone else's opportunity (to *X*) in the relevant way (e.g., by rendering physically impossible the doing of *X* (if someone tried to *X*)) (Kramer, 2003: 133–135).

^16^
Notice that this also follows from holding that freedom has content‐independent value (Kramer, 2017: 200–207), and that therefore *any* freedom removal (independently of the content of the freedom interfered with) is disvaluable.

^17^
On this interpretation of a pro tanto reason, I follow Richard Rowland (2019: chap. 1). My interpretation is distinct from one that contrasts “pro tanto” with “prima facie”: on the latter interpretation, pro tanto refers to a duty that remains a duty even if it can be overridden in certain circumstances, whereas prima facie refers to “a rebuttable presumption” (Kramer, 2017: 157) in favour of (refraining from) *X* that counts as a duty only *at first glance* (prima facie), that is, only when it is not overridden. I adopt the simpler interpretation that equates pro tanto reasons with contributory reasons (without distinguishing between pro tanto and prima facie) because it is better suited to the language of reasons that I adopt to analyse The Status View.

^18^
If the value of the freedom to *X* is entirely dependent on the value of *doing X*, there would be no value in the freedom to *X* in all those cases in which doing *X* (like walking into the quicksand) is objectively disvaluable.

^19^
To be precise, Kramer's claim is that *increases* in one's degree of freedom *enhances* “the declinatory force of a person's choices” (Kramer, 2017: 204). But it is also true that freedom removals have the reverse effect.

^20^
Note that not just human interferences (i.e., constraints in the relevant sense attributable to human beings) but also obstacles that have a natural origin (e.g., physical disabilities, natural catastrophes) can negatively affect one's opportunities for agency. But whether also natural obstacles should count as sources of unfreedom, in my view, depends on which kind of value is attributed to freedom (and disvalue to unfreedom) since I endorse a value‐first approach to the definition of freedom/unfreedom. And, as I argue in §5, insofar as one interprets the value of freedom in relational and deontic terms, one has reason to hold that only human interferences can generate unfreedom. That is, one can think that natural constraints can negatively affect one's opportunities for agencies while also holding that this is irrelevant for the purpose of defining unfreedom (on grounds of endorsing a relational account of the value of freedom grounded on deontic reasons).

^21^
It is unclear whether Kramer interprets this type of value only in axiological terms (i.e., by directly appealing to the good) (this seems implied by Kramer's remarks at pp. 251–253, where Kramer reflects on the arguments put forward in chap. 5) or also in deontic terms (which seems implicit to Kramer's reference to “respect for individual citizens and their agency” at p. 251). My analysis clarifies the deontic interpretation, upon which I ground The Status View.

^22^
This conclusion on the impersonal value of freedom (as robust non‐interference) is reached also by Pettit (2015: 202–204), who discusses personal versus impersonal reasons to value freedom (and the goods of attachment, virtue, and respect) (Pettit, 2015: chap. 7).

^23^
To the extent that it is valuable that *B* has freedom, *A* has *some* reason to refrain from relevantly interfering with *B*'s freedom; yet, qua agent, *A* may also have *some* reason to exercise her agency in ways that deprive *B* of the freedom to *X*. For example, although in *Quicksand* Mildred has some reason to refrain from depriving Noah of the freedom to enter the quicksand, she also has some reason to deprive Noah of such a freedom (since achieving her artistic aims would entail relevantly interfering with Noah's freedom).

^24^
Although in this section I have shown that there are several accounts of the relationships between the value of freedom and agency, in the following sections I single out the one expressed by The Status View because I am interested in showing the possibility of appealing to freedom's value in a theory of justice without appealing to well‐being, and of interpreting the definition of freedom in light of The Status View. What grounds assuming a deontological approach to justice is a more fundamental question that is beyond the scope of this paper. I thank an anonymous reviewer for asking to clarify this.

^25^
As I clarify below “non‐specific” and “content‐independent” value capture the same aspect of the value of freedom.

26 This point is made also by Bader (2018) at p. 159 (see quotation below). Bader defends a moralised conception of freedom, according to which only the opportunities to perform morally permissible actions should count as freedoms. And he explains the value attributable to moralised freedom in the following terms: “Given that rights are deontic notions, the best way to think of the normative significance of moralized freedom is not axiologically in terms of the goodness of the resulting opportunities, but deontically in terms of the wrongness of interference. The reason why being free to *ϕ* matters is not because one thereby has the opportunity to *ϕ*, but rather because one's right to *ϕ* is being respected and because one is not illegitimately being prevented from *ϕ*‐ing. Any illegitimate interference with what one has a right to do is intrinsically unjust and disrespectful of one's moral status. By contrast, the opportunity to *ϕ* is at best derivatively good, and moreover its being good presupposes that the opportunity is a real as opposed to a merely formal opportunity.”

^27^
By “respect” I mean the attitude of refraining from relevantly interfering with people's freedom. Most plausibly, respect‐as‐attitude is also a component of the moral principle of respect for persons (e.g., Bader, 2018: §4; Carter, 2009; Lawless, 2018; Pettit, 2015: chap. 3).

^28^
Asserting non‐specific/content‐independent value does not deny that part of the value of freedom is *specific* or *content‐dependent*: that freedom (also) has value in virtue of its content (qua freedom to *X*; *Y*; *Z*) (Kramer, 2003: 240–245). For instance, the freedom to eat vegetables has both content‐dependent value (if performing what such a freedom allows to do is valuable) and content‐independent value.

^29^
As Kramer claims in commenting on *Desires*: “[s]ince nobody at present can infallibly identify the particular freedoms that are most beneficial for us, our lives will generally go better if our combinations of conjunctively exercisable freedoms are plentiful rather than meager” (Kramer, 2017: 206).

^30^
For Bader such opportunities (to perform severely wrong actions) should not be called “freedoms” since on Bader's moralised account only unobstructed opportunities to perform morally *permissible* actions should count as ‘freedoms.’

^31^
Thereby also depriving the individual of the opportunity to do so.

^32^
For context on ideal theory, see Stemplowska and Swift (2012).

^33^
Pettit has recently developed a theory of interpretation for the definition of concepts (Pettit, 2020). In the final section of the paper, Pettit applies his theory to issues concerning the definition of freedom (Pettit, 2020: §5). I was not aware of Pettit's contribution while writing this paper, and my contribution converges with Pettit's analysis in holding that the choice between different definitions of freedom is “to be made on independent, non‐analytical grounds” (Pettit, 2020: 353).

^34^
More specifically, definitions fit with value‐based considerations when such considerations give reason to define the concepts in question (e.g., freedom) one way or another. I do not assume that such considerations *determine* or *necessarily lead to* endorsing a certain definition: the interpreter does not necessarily need to show that a relationship of entailment subsists between value‐based considerations and a given definition of a concept. On a weaker account, it suffices that such considerations *count in favour of* or *are conducive to* endorsing the definition in question.

^35^
I thank an anonymous reviewer for posing this question.

^36^
In other words, I shall not take a stance on whether all values are connected in an integrated network through mutually reinforcing justificatory relations (Dworkin's unity of value thesis).

^37^
Hence, I leave aside which considerations concerning the definition of freedom would follow if one appeals (also) to the good in a theory of justice, and whether it is possible to find convergence on a definition of freedom that is coherent both with reasons concerning the good and with deontic reasons.

^38^
I understand ability in terms of likelihood of success in doing *X*, if one tries to *X* (Hindriks, 2008: 166–167).

^39^
Relational freedom theorists disagree on whether the intervening agent must be *causally* (Kramer, 2003: chap. 4) or *morally* (Miller, 1983) responsible.

^40^
Relational freedom can be combined with an ability‐based definition of freedom: indeed, one can consistently hold both that only constraints relevantly attributable to other human beings are unfreedom‐generating, and that, in order to be free to *X*, one should be able to *X* (in the absence of the relevant human constraints) (Kramer, 2003).

^41^
That the definition of freedom that fits with The Status View is one that interprets freedom as *formal* opportunities (i.e., mere absence of interference) is emphasised also by Bader (2018: §4). Of course, as I clarify in §3, there can be alternative (i.e., non‐relational) accounts of the relationship between freedom and agency. Here I assume the one singled out by The Status View.

^42^
For example, John Lawless observes that for theories of “negative liberty” – which in the present context can be equated with what I call *Relational Freedom* – “the special significance of the interpersonal manifests in the kinds of constraints that make a person unfree” ((Lawless, 2018: 2) (i.e., in the fact that according to such theories only human constraints can be considered as unfreedom‐generating)) (see Lawless, 2018: §2; Pettit, 2003: 396). Lawless grounds the focus on “the interpersonal” on a more fundamental interest in respect as status recognition (Lawless, 2018: §2) but then argues that theories of negative freedom ultimately fail to properly account for respect (Lawless, 2018: §3).

^43^
For Steiner, the compossibility of rights is a logical requirement of any plausible set of rights, and this justifies understanding rights as negative rights. Negative rights entitle to other people's forbearances from acting, and therefore never conflict. Hence, since Steiner defines freedom as mere absence of relevant human interferences, he can claim that (negative) “rights prescribe interpersonal distributions of pure negative liberty” (Steiner, 1994: 57).
